# New Coordination Complexes Based on the 2,6-bis[1-(Phenylimino)ethyl] Pyridine Ligand: Effective Catalysts for the Synthesis of Propylene Carbonates from Carbon Dioxide and Epoxides

**DOI:** 10.3390/molecules23092304

**Published:** 2018-09-10

**Authors:** Li Xia, Wen-Zhen Wang, Shuang Liu, Xin-Gang Jia, Ying-Hui Zhang, Lei-Lei Li, Ya Wu, Bi-Yun Su, Shu-Bo Geng, Wei Fan

**Affiliations:** 1 School of Chemistry and Chemical Engineering, Xi’an Shiyou University, Xi’an 710065, China; lxia9264@163.com (L.X.); liush@xsyu.edu.cn (S.L.); jiaxingang76@xsyu.edu.cn (X.-G.J.); lll@xsyu.edu.cn (L.-L.L.); wuya@xsyu.edu.cn (Y.W.); subiyun@163.com (B.-Y.S.); vfan111@163.com (W.F.); 2 Department of Chemistry, Nankai University, TianJin 300071, China; zhangyhi@nankai.edu.cn (Y.-H.Z.); shubogeng216@163.com (S.-B.G.)

**Keywords:** coordination complexes, carbon dioxide, catalyst for propylene carbonate, DFT calculations

## Abstract

We aimed to develop new effective catalysts for the synthesis of propylene carbonate from propylene oxide and carbon dioxide. A kind of M^x+^**L**Cl_x_ coordination complex was fabricated based on the chelating tridentate ligand 2,6-bis[1-(phenylimino)ethyl] pyridine (**L**). The obtained products were characterized by elemental analysis, infrared spectroscopy, ultraviolet spectroscopy, thermogravimetric analysis, and single-crystal X-ray diffraction. It was found that the catalytic activity of the complexes with different metal ions, the same ligand differed and co-catalyst, where the order of greatest to least catalytic activity was **2** > **3** > **1**. The catalytic system composed of complex **2** and DMAP proved to have the better catalytic performance. The yields for complex **2** systems was 86.7% under the reaction conditions of 100 °C, 2.5 MPa, and 4 h. The TOF was 1026 h^−^^1^ under the reaction conditions of 200 °C, 2.5 MPa, and 1 h. We also explored the influence of time, pressure, temperature, and reaction substrate concentration on the catalytic reactions. A hypothetical catalytic reaction mechanism is proposed based on density functional theory (DFT) calculations and the catalytic reaction results.

## 1. Introduction

Recently, considerable attention has been paid to the fixation of carbon dioxide, due to its key role as a greenhouse gas. The chemical fixation and conversion of carbon dioxide into valuable chemicals is generally regarded as an excellent method from both the environmental protection and resource utilization standpoints [1,2,3,4,5]. As a naturally abundant, cheap, recyclable, and nontoxic carbon source, carbon dioxide has been involved in various organic reactions, especially the cycloaddition of epoxides with carbon dioxide to produce carbonates [6,7,8,9,10,11]. This reaction is a standard “atom-economy” and “green-chemistry” reaction to produce environmentally-friendly propylene carbonate that is widely utilized as a special solvent in many chemical industries such as liquefied natural gas (LNG), the textile printing industry, lithium batteries, and wood processing [12,13,14,15]. Many methods have been developed for the synthesis of propylene carbonate, including urea alcoholysis, phosgene (carbonyl chloride), chloropropanols, cycloaddition, and ester exchange. Among these methods, ester exchange is the simplest and easiest one to operate, with low cost, high product yield, good selectivity, and high quality [16,17,18]. However, the reaction conditions are harsh, and the mechanism of the catalytic reaction remains unclear. In the 1990s, Kruper [19] reported the synthesis of cyclic carbonates from epoxides and CO_2_ catalyzed by metalloporphyrins. To improve the catalytic activity of metal complexes, a salen ligand was used to replace the porphyrin ligand, and higher catalytic performance was observed as expected [20]. In 2000, Kim et al. [21,22,23] reported the synthesis of propylene carbonate catalyzed by zinc complexes. Similarly, Shi’s group [24] found that the catalytic systems comprising a Schiff base/organic base and phenol/organic base components exhibit high activity in catalyzing the synthesis of cyclic propylene carbonate from CO_2_. In 2017, ojas’s group developed a series of amidinate aluminium complexes as catalysts for the conversion of carbon dioxide into cyclic carbonates [25].

**Scheme 1:**
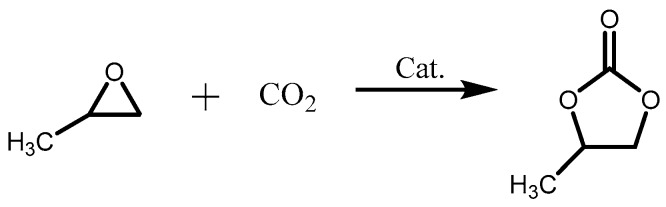
The synthesis of propylene carbonate (PC) from carbon dioxide and propylene oxide.

In this work, three metal complexes based on 2,6-bis[1-(phenylimino)ethyl] pyridine (**L**), namely [Cu**L**Cl_2_] (**1**), [Cr**L**Cl_3_] (**2**), and [Mn**L**Cl_2_]·0.5(CH_3_CN) (**3**) were constructed, and were investigated in terms of their activity in catalyzing the cycloaddition reaction between propylene oxide and carbon dioxide (Scheme 1) [26,27,28], in the presence of co-catalyst. Furthermore, the relationship between the molecular structures and catalysis performance was analyzed based on experiment and density functional theory (DFT) calculation, and a reaction mechanism is proposed.

## 2. Results and Discussion

### 2.1. Crystal Structures

**Figure 1:**
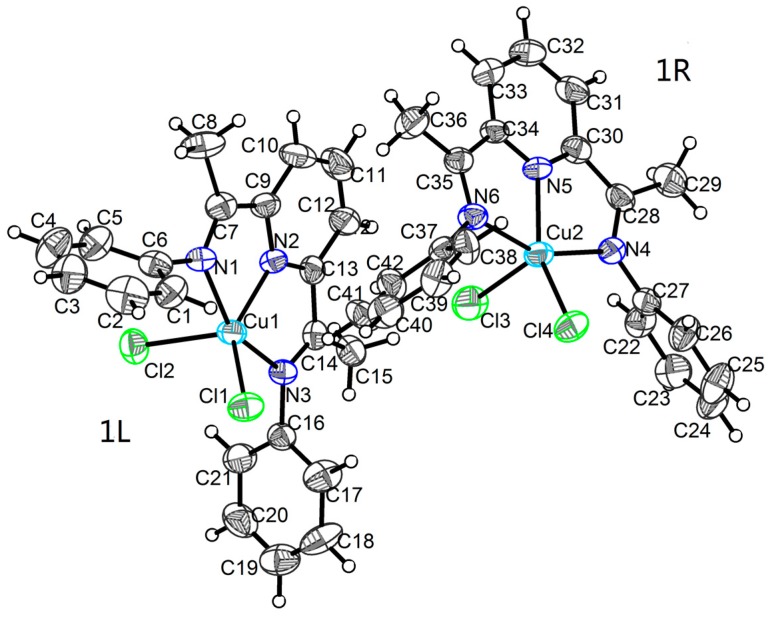
The molecular structure and crystallographic numbering scheme for complex **1**.

**Figure 2:**
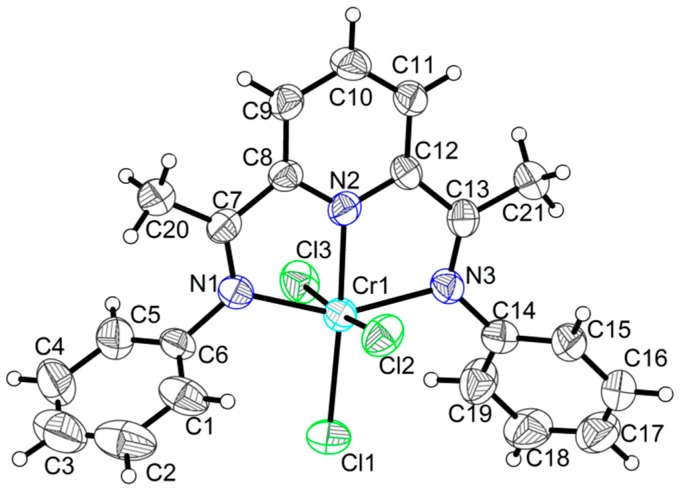
The molecular structure and crystallographic numbering scheme for complex **2**.

**Figure 3:**
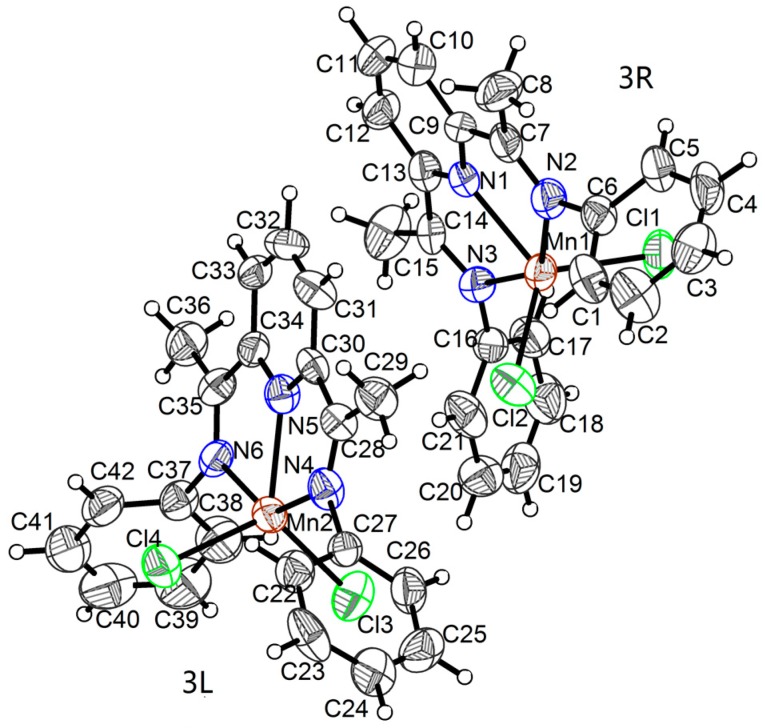
The molecular structure and crystallographic numbering scheme for complex **3** (CH_3_CN molecules are omitted for clarity).

Complexes **1**–**3** were analyzed by single-crystal X-ray crystallography (see Figure 1, Figure 2 and Figure 3). The relevant crystallographic and structural modification data are collected in Table 1, and partial data/parameters of the bond lengths and bond angles are summarized in Table 2. Complex **1** is shown in Figure 1, crystallized in the orthogonal system space group, *P*bca, with *Z* = 16 and with two independent molecules (**1**L and **1**R) in the asymmetric unit. The molecules (**1**L and **1**R) all contain one Cu(II) ion, one bis(imino)pyridine ligand, and two coordinated chloride ions. The Cu(II) adopts a pentacoordinated structure, in which one pyridine nitrogen atom and two chlorine atoms form the basal plane (N2, C11 and C12) (or (N5, C32 and C33)) and two imino-nitrogen atoms occupy the apical position with a N1–Cu–N3 (or (N4–Cu2–N6)) angle of 155.48(18)° (or 154.78(17)°). The calculated value of the τ factor [29,30] for Cu1 is 0.23, indicating a significant distortion of the metal surroundings from square pyramidal (τ = 0) toward trigonal bipyramidal (τ = 1). For Cu2, this value is 0.09. Thus, the **1**L and **1**R molecules all are distorted square pyramidal geometry, and the **1**R molecule is nearer to perfectly square pyramidal than the **1**L molecule. Although systems are unsuitable, the result can be used a reference to roughly explain the degree of distortion for the molecules. The dihedral angles between the three nitrogen-coordination plane and two phenyl rings are 70.97° (C1–C6) and 87.39° (C16–C21), respectively. For molecule **1**R, they are 59.78° (C22–C27) and 88.27° (C37–C42), respectively. Meanwhile, these two phenyl rings are nearly perpendicular with a dihedral angle of 85.35° and 89.99° for **1**L and **1**R. The central metal copper atoms deviate from the three nitrogen-coordination planes by all about 0.001 Å for **1**L and **1**R and deviate from the equatorial plane by about 0.006 Å and 0.005 Å for **1**L and **1**R, respectively. In both **1**L and **1**R, the lengths of the Cu–N_imino_ bonds are all longer than the Cu–N_pyridyl_ bond. Moreover, N1–C7 (1.273(6) Å), N3–C14 (1.295(7) Å), N4–C28 (1.284(6) Å) and N6–C35 (1.273(6) Å) are of typical double bond character.

Complex **2** crystallizes in the tetragonal system, space group *I*4_1_/*a* with *Z* = 16, and the crystal structure is shown in Figure 2 The molecular structure contains one Cr (III) ion, one bis(imino)pyridine ligand, and three coordinated chloride ions. The Cr (III) adopts a six-coordinated octahedral structure, in which there is one pyridine nitrogen atom, three chlorine atoms, and two imino-nitrogen atoms. The dihedral angles between the three nitrogen-coordination planes and two phenyl rings are 68.77° (C1–C6) and 49.17° (C14–C19), respectively. Meanwhile, these two phenyl rings are nearly perpendicular, with a dihedral angle of 86.94°. The central metal copper atoms deviate from the three nitrogen-coordination planes and the equatorial plane by about 0.047 and 0.177 Å, respectively. The lengths of Cr–N_imino_ bond (2.109(5) and 2.111(5) Å) are longer than Cr–N_pyridyl_ bond (2.001(5) Å). Moreover, the N1–C7 (1.281(7) Å) and N3–C13 (1.295(7) Å) bond lengths show typical double bond character.

Complex **3** crystallizes in the triclinic system, space group *P*$\bar{1}$ with *Z* = 4 and with two independent molecules (**3**L and **3**R) and an acetonitrile molecule in the asymmetric unit, as shown in Figure 3. The molecules (**3**L and **3**R) all contain one Mn (II) ion, one bis(imino)pyridine ligand, and two coordinated chloride ions. Like complex **1**, the Mn (II) adopts a pentacoordinated structure, in which one pyridine nitrogen atom and two chlorine atoms form the basal plane (N1, C11 and C12) (or (N5, C32 and C33)) and two imino-nitrogen atoms occupy the apical position with a N2–Mn–N3 (or (N4–Cu2–N6)) angle of 143.30° (or 154.78(17)°).

The Mn1 and Mn2 have a τ value of 0.28 and 0.25, respectively. Thus, the **3**L and **3**R molecules are all of distorted square pyramidal geometry, and their distortion levels are approximately the same. The dihedral angles between the three nitrogen-coordination planes and two phenyl rings are 65.17° (C1–C6) and 71.39° (C16–C21), respectively. For molecule **3**R, they are 59.78° (C22–C27) and 88.27° (C37–C42), respectively. Meanwhile, these two phenyl rings are nearly perpendicular with a dihedral angle of 75.56° and 89.63° for **3**L and **3**R. The central metal copper atoms deviate from the three nitrogen-coordination planes and the equatorial plane by about 0.016 Å and 0.017 Å, respectively. For molecule **3**R, these values are 0.015 Å and 0.016 Å, respectively. Molecules **3**L and **3**R both have Mn–N_imino_ bond lengths greater than the length of the Mn–N_pyridyl_ bond. Moreover, N2–C7 (1.270(16) Å), N3–C14 (1.296(11) Å), N4–C28 (1.280(10) Å) and N6-C35 (1.293(10) Å) have typical double bond character.

### 2.2. Cycloaddition of CO_2_ and Propylene Oxide

The cycloaddition of propylene oxide (PO) to CO_2_ was conducted in the presence of various catalysts, and the results are listed in Table 3. No reaction was observed in the absence of co-catalyst and the presence of only 4-dimethylaminopyridine (DMAP) (entries 1 and 5, Table 3), which is consistent with that observed for some previous reports [31]. However, the reaction occurred when TBAB alone presented. The phenomenon mainly stems from the fact that TBAB, as a quaternary ammonium salt, is an effective catalyst for the catalytic synthesis of carbonates from carbon dioxide and alkylene oxides [32,33]. Interestingly, complexes **1**–**3** demonstrated excellent catalytic performance with both co-catalysts for the synthesis of propylene carbonate (entries 2–4 and 7–8, Table 3). The efficiency of complexes **1**–**3** decreased in the order **2** > **3** > **1**. The highest turnover frequency (TOF) value reached 373 h^−^^1^ under mild conditions for complex **2**. Moreover, complex **2**/TBAB all showed well catalytic performance, the TOF value reached 348 h^−^^1^. Liu et al. [34] reproted aluminum (salen) complexes as catalyst for the synthesis of propylene carbonate, and the TOF was as low as 189 h^−^^1^. The chromium-salen complexes of Ramin et al. [35] showed propylene carbonate TOF of 90–330 h^−^^1^ at 140 °C. Complex **1** showed the lowest catalytic performance among complexes **1**–**3**, but its TOF still reached 204 h^−^^1^, which is quite effective.

To further optimize the reactivity of this catalyst system, the effect of various reaction parameters on PC formation was investigated using complex **2**/DMAP as catalyst. Representative results are summarized in Table 4. The coupling reactions were performed under pressures ranging from 2.0 MPa to 3.5 MPa at 120 °C. As shown in Table 4, the pressure apparently influenced the PO and CO_2_ cycloaddition reaction at low pressures. The reaction could be realized at 2.0 MPa with a TOF of 337 h^−1^, and the yield increased by 15.5% as the pressure changed from 2.0 MPa to 3.0 MPa (entry 1 vs. entry 3, Table 4). With further increases in the operating pressure above 3.0 MPa, the yield of the product increased slightly (entry 3 vs. entry 4, Table 4). For practical reasons, the following experiments were conducted at a fixed pressure of 2.5 MPa.

The effect of the molar ratio of PO to complex **2** on the reaction was evaluated under otherwise identical reaction conditions. The data in entries 2, 8, 11, and 12 in Table 4 show that the yield of PC decreased markedly from 74.6% to 14.8% with increasing monomer usage. Regarding the effect of reaction time (entries 5–7, Table 4), a prolonged reaction time was found to be beneficial for improving the PC yield. In terms of the TOF, the appropriate reaction time was 3 h, in which the TOF reached 554 h^−1^ (entries 6 and 7, Table 4). The ratio of DMAP to complex **2** had a significant effect on the TOF (entries 5, 13, and 14, Table 4) and showed the TOF increased as the number of equiv. of DMAP increased, up to two equiv. (entries 5 and 13, Table 4). Increasing the DMAP concentration any further resulted in a loss of activity, to the point where the reaction was almost completely shut down when 4 equiv. of DMAP were used (entry 14, Table 4). Entries 5, 8, 9, and 10 in Table 4 demonstrate the effect of temperature on the reaction efficiency. The catalytic activity was clearly sensitive to temperature. The PC yield appeared to decrease as the temperature increased from 200 °C to 220 °C, probably because of catalyst decomposition at higher temperatures. In fact, we found that complex **2** began to break down at temperatures above 220 °C, as determined by thermogravimetric analysis. (see Figure S1 in Supplementary Materials).

### 2.3. Mechanism for the Cycloaddition Reaction of Propylene Oxide and CO_2_

**Scheme 2:**
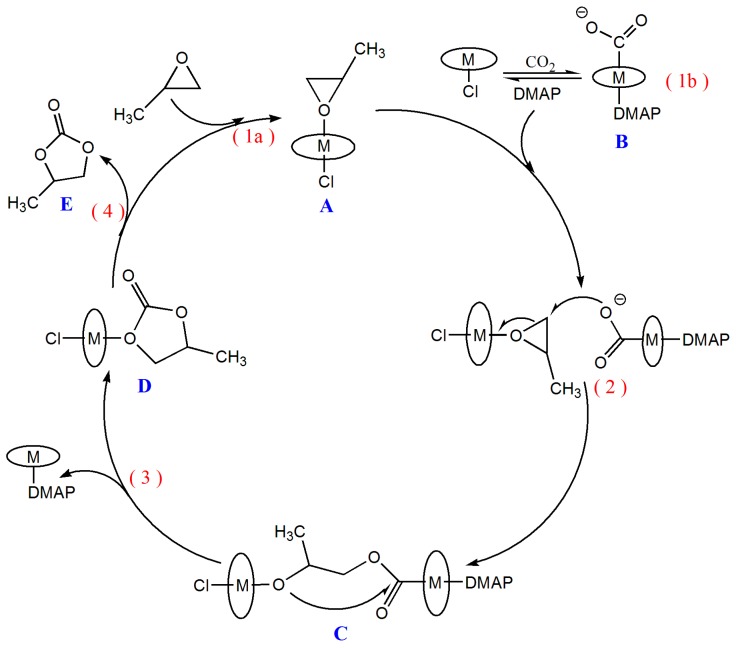
Proposed mechanism for the cycloaddition of propylene oxide to CO_2_ with DMAP.

Jacobsen et al. have shown that the nucleophilic ring opening of epoxides catalyzed by Cr(III) salen complexes occurs via the catalyst activation of both the electrophilic epoxide and the incoming nucleophile [36]. Previous reports on the synthesis of cyclic carbonate from CO_2_ and epoxides also suggest the parallel requirement of both Lewis base activation of the CO_2_ and Lewis acid activation of the epoxide [37,38]. According to the above catalytic reaction result, one possible coordination-insertion reaction mechanism is proposed as shown in Scheme 2 [31,39]. Based on the ring-catalyzed reaction mechanism, the alkylene oxide was activated by complexes (**1a**), and species **A** was formed. At the same time, the DMAP co-catalyst joined in the formation of species **B**, which related to the activation of CO_2_ (**1b**). Subsequently, species **B** can then attack the activated epoxide species **A** at the least sterically hindered carbon (**2**), leading to the formation of the dimeric intermediate **C** which eventually yields the cyclic carbonate product **E** (**3** and **4**). This mechanism can explain the dramatic losses in activity that accompany an increase, beyond an optimal concentration, in either DMAP molar equivalents or CO_2_ pressure. Both of these situations would lead to an increased formation of **B** at the expense of **A**, thereby reducing the reaction rate.

**Figure 4:**
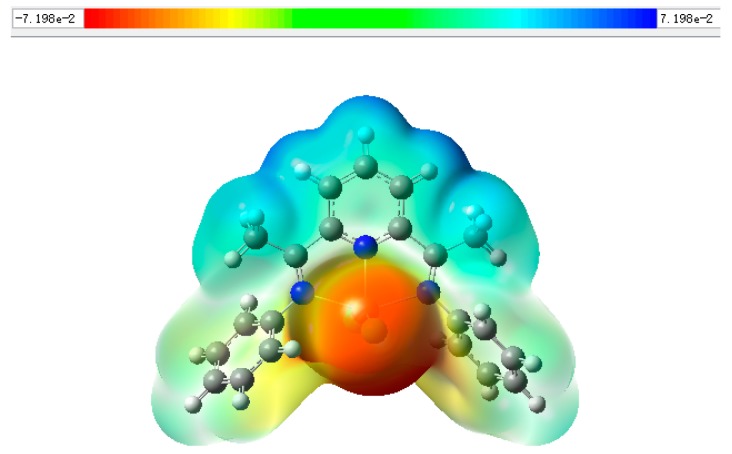
The electrostatic potential (ESP) of complex **1**.

**Figure 5:**
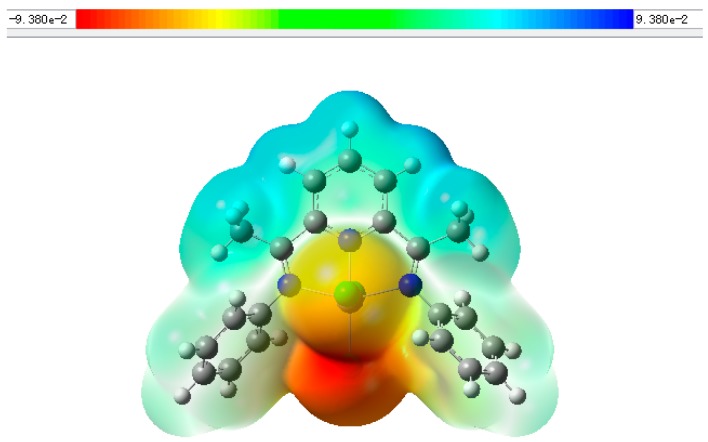
The electrostatic potential (ESP) of complex **2**.

**Figure 6:**
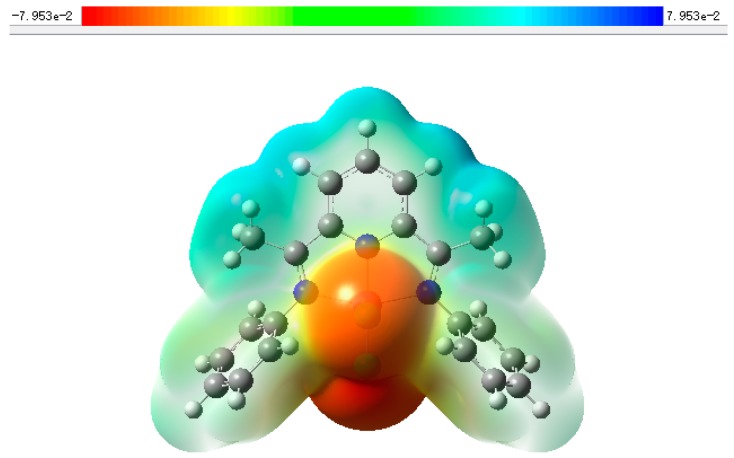
The electrostatic potential (ESP) of complex **3**.

In order to further explore the influencing factors of the reaction mechanism, DFT calculations [40] were performed on the three complexes. The frontier molecular orbitals of complexes **1**–**3**, including the highest occupied molecular orbital (HOMO) and the lowest unoccupied molecular orbital (LUMO), are shown in Figures S4–S6 (see Supplementary Materials), respectively. The negative HOMO orbital energy values indicate that the electronic state of the complex was stable. The natural bond orbitals (NBO) charges [41] for selected atoms are listed in Table S1 (see Supplementary Materials). The segmental results of calculations are shown in Table 5 and the proprietary results of calculations are shown in Table S2 (see Supplementary Materials). The electrostatic potential (ESP) [42] of complexes **1**–**3**, as shown in Figure 4, Figure 5 and Figure 6, respectively. It is apparent that the electrostatic potential surface of the metal ions was influenced by the charge density distribution. The positive charge on the surface of complex **2** was stronger than those on complexes **3** and **1**. It is consistent for catalytic reaction results (entries 2, 3 and 4, Table 3), which indicating that the stronger the acidity of Lewis acid, the higher catalytic reaction will be [43]. Moreover, Table 5 also reveals that the energy gap ΔE values (ΔE = E_LOMO_ − E_HOMO_) of the three complexes followed the order from highest to lowest: **2** < **3** < **1**. Since complex **2** had the lowest energy gap ΔE, catalytic reaction occurred relatively easily for this system.

Some conclusions are obtained as follows according to the catalytic reaction results, DFT calculations and mechanism. First, the larger the net charge of metal ions are, the easier the nucleophilic attack on oxygen atom to activate propylene oxide (see **1a** in Scheme 2). On the contrary, it is easier for electrophilic attack on carbon atom to activate carbon dioxide (see **1b** in Scheme 2). Therefore, only the suitable charged metal in complexes can join in both procedures (**1a** and **1b**) and give rise to the catalytic activation. Second, the net charge of metal ions are not decisive factor, the energy gap ΔE values (ΔE = E_LOMO_ − E_HOMO_) also play a important role that smaller ΔE values are, the higher catalytic reaction activation (see Table 5). Third, the activation of carbon dioxide is the decisive step for the catalytic reaction probably. Fourth, by properly fine-tuning the charge of the active metal center it is possible to develop a highly active catalyst.

## 3. Materials and Methods

### 3.1. Chemical Materials

2,6-Diacetylpyridine, aniline, copper (II) chloride dihydrate (CuCl_2_·2H_2_O), anhydrous chromium (III) chloride (CrCl_3_), and manganese (II) chloride tetrahydrate (MnCl_2_·4H_2_O) were obtained from commercial sources and used without further purification unless otherwise noted.

### 3.2. X-ray Crystallographic Studies

Single crystals with proper dimensions were selected for single-crystal X-ray diffraction measurement, and the relevant diffraction data were collected on an APEX CCD II Bruker single crystal diffraction meter (Bruker, Billerica, MA, USA). At 296(2) K, 38290, 20559, and 11205 X-ray reflections were collected, respectively, for complexes **1**, **2**, and **3** using graphite monochromated Mo K*α* (*λ* = 0.71073 Å) and *φ-ω* scan mode. The crystal structures were refined using SHELXL-97 (University of GÖttingen: GÖttingen, Germany) [44] analytical procedures, while the coordinates of the non-hydrogen atom structure and anisotropic parameters were obtained using the full matrix least square method in the SHELXL-97 program. The CCDC deposition numbers of the complexes are 1508337 (**1**), 1496950 (**2**), and 1860911 (**3**), respectively.

The following restrains were introduced to improve the quality of the crystal data. For complex **1**: (1) the phenyl ring defined by C16 > C21 was restrained by AFIX 66 instruction to give rational C-C bond lengths; (2) Delu 0.01 N2 C7 instruction was used to confirm the two bonded atoms have rational parameters. For complex **3**: Alert level B suggested the structure exist a large error at low angle diffraction point, we find the bad point related HKL (0 0 2) in the “most disagreed reflection” in the .lst file, then add “omit 0 0 2” to the .ins file, and refine it.

### 3.3. Catalyst Characterization

The infrared data of the complexes were recorded on a VERTEX-70 Fourier transform infrared spectrometer with a band range of 4000–400 cm^−1^, where the measured samples were dealt with KBr tablet. The elemental analysis data of complexes were obtained by using a Vario MICRO element analyzer from Elementar (Langenselbold, Germany). The thermogravimetric analysis data of complexes were obtained using a Mettler Toledo thermogravimetric analysis system (Mettler Toledo, Columbus, OH, USA). The UV spectrum data of complexes were obtained by using a UV-2600 ultraviolet spectrophotometer (Shimadzu, Kyoto, Japan).

### 3.4. Catalyst Preparation

#### 3.4.1. Synthesis of the Ligand (2,6-bis[1-(Phenylimino)ethyl] pyridine) (**L**)

2,6-Diacetylpyridine (0.50 g, 3.06 mmol) and aniline (0.095 g, 1.02 mmol) were added to 100 mL methanol. After the addition of several drops of formic acid, the reaction mixture was refluxed for 12 h at 40 °C. A yellow powder was obtained by the filtration of crude yellow precipitate, evaporation of the solvent and recrystallization from methanol. The yield was 70%. EA (%) C_21_H_19_N_3_: calcd. C 80.40, H 6.06, N 13.40; found: C 80.36, H 6.19, N 13.31; UV-Vis (CH_2_Cl_2_) λ_max_/nm (ε/dm^3^ mol^−1^ ·cm^−1^) = 285 (6.39 × 10^4^), 249 (1.10 × 10^5^); IR (KBr, cm^−1^): 2981 (m), 1653 (vs), 1559 (m), 1492 (s), 1468 (s), 1407 (s), 1319 (m), 1217 (s), 1116 (m), 1071 (s), 955 (w), 932 (m), 872 (m), 820 (s), 770 (w), 740 (m), 633 (w), 547 (m), 451(w).

#### 3.4.2. Synthesis of Complexes

[Cu**L**Cl_2_] complex **1**: The ligand **L** (0.116 g, 0.37 mmol) and CuCl_2_·2H_2_O (0.063 g, 0.37 mmol) were dissolved in 30 mL CH_3_CN, which were stirred for 12 h at room temperature under the protection of nitrogen. Then, a deep yellow solid powder was obtained after distillation of CH_3_CN solvent. Finally, deep yellow crystals were gained after the recrystallization with CH_3_CN, with a yield of 76%. EA (%) C_21_H_19_Cl_2_CuN_3_: calcd. C 56.27, H 4.24, N 9.38; found: C 56.19, H 4.33, N 9.42; UV-Vis (CH_2_Cl_2_) λ_max_/nm (ε/dm^3^ mol^−1^·cm^−1^) = 284 (5.06 × 10^4^), 246 (9.05 × 10^4^); IR (KBr, cm^−1^): 3436 (m), 2919 (m), 1637 (vs), 1485 (m), 1384 (s), 1270 (m), 1233(s), 1209 (m), 872 (m), 820 (s), 692 (m), 633 (w).

[Cr**L**Cl_3_] complex **2**: The synthetic method for complex **2** was similar to that of complex **1** except for the replacement of CuCl_2_·2H_2_O by anhydrous CrCl_3_ (0.059 g, 0.37 mmol). Light green crystals were obtained with a yield of 79%. EA (%) C_21_H_19_Cl_3_CrN_3_: calcd. C 53.45, H 4.03, N 8.91; found: C 53.38, H 4.18, N 8.87; UV-Vis (CH_2_Cl_2_) λ_max_/nm (ε/dm^3^ mol^−1^·cm^−1^) = 246 (8.24 × 10^4^); IR (KBr, cm^−1^): 2925 (s), 2851 (s), 1637 (vs), 1462 (s), 1383 (s), 1304 (m), 1256 (m), 1231 (w), 1042 (w), 859 (w), 823 (w).

[Mn**L**Cl_2_]·0.5(CH_3_CN) complex **3**: The synthetic method for complex **3** was similar to that of complex **1** except for the replacement of CuCl_2_·2H_2_O by MnCl_2_·4H_2_O (0.073 g, 0.37 mmol). Orange crystals were obtained with a yield of 71%. EA (%) C_2__2_H_20.5_Cl_2_MnN_3__.5_: calcd. C 57.39, H 4.46, N 10.65; found: C 57.27, H 4.42, N 9.58; UV-Vis (CH_2_Cl_2_) λ_max_/nm (ε/dm^3^ mol^−1^·cm^−1^) = 280 (1.71 × 10^5^), 246 (2.25 × 10^5^); IR (KBr, cm^−1^): 2918 (m), 2789 (m), 1627 (vs), 1593 (vs), 1485 (s), 1371 (m), 1257 (m), 1225 (s), 1169 (m), 1018 (s), 818 (w), 777 (m), 721 (s), 695 (w), 631 (w).

#### 3.4.3. Catalytic Procedure

The detailed catalytic procedure was described in our previous work [45]. A 100 mL stainless-steel reactor was charged with purified propylene oxide (PO) and catalyst. Then, the reactor was pressurized to desired pressure with carbon dioxide and heated to the desired temperature by stirring. After predetermined time of reaction, the autoclave was cooled down to room temperature, and the CO_2_ pressure was released by opening the outlet valve. The solid residue was separated from the reaction mixture by filtration. The product propylene carbonate (PC) was obtained through distillation of the filtrate under reduced pressure. Finally, a qualitative analysis of the liquid products was performed on FT-IR spectrum in Figure S5 (see Supplementary Materials). For quantitative determination, the products were analyzed on an Agilent 6890 Plus GC with flame ionization detection. The PC yield was obtained by internal standard method (Biphenyl as the internal standard substance).

### 3.5. Gaussian Calculation

All DFT calculations in this work were performed with the B3LYP functional by using the Gaussian 09W (Gaussian, Inc., Wallingford, CT, USA) program. The effective nuclear LanL2DZ basis set was adopted for metal atoms, and the 6-31G (d, p) [46] basis set for other atoms. **1**L and **3**R were used as calculation models for complexes **1** and **3**, respectively. It was found that the optimized geometric structure was substantially similar to the experimental one. Partial predicted bond lengths and bond angles are collected in Table S2 (see Supplementary Materials). Frequency calculations at the same theoretical level confirmed that the optimized structures settled at a local minimum site of the potential energy surface. The atomic charge distributions were calculated by natural bond orbital (NBO) analysis to better understand the catalytic activity.

## 4. Conclusions

In this work, three different metal complexes **1**–**3** were synthesized from the selected tridentate nitrogen-containing ligand 2,6-bis[(1-phenylimino)ethyl] pyridine (**L**). It is worth highlighting that these widely used metal complexes for ethylene oligomerization were applied for the first time to the synthesis of cyclic propylene carbonate from propylene oxide and carbon dioxide. The catalytic activity of the complexes was evaluated in the synthesis of the propylene carbonate from propylene oxide and carbon dioxide. The catalytic investigation revealed outstanding catalytic activity for [Cr**L**Cl_3_] (**2**), with propylene carbonate (PC) yields >80% in the presence of 4-dimethylaminopyridine (DMAP), outperforming complexes [Cu**L**Cl_2_] (**1**) and [Mn**L**(CH_3_CN)_0.5_Cl_2_] (**3**). A supposed catalytic reaction mechanism is proposed based on the DFT calculations and catalytic reaction results. It is a competitive relationship between the activation of carbon dioxide (**1b**) and propylene oxide (**1a**), which the former one could have a greater inflence for catalytic reaction. Moreover, the energy gap ΔE values can reveal catalytic activation very intuitively. This work provides a simple method for future research.

## Figures and Tables

**Table 1:** Crystal data for complexes **1**–**3**.

Compound	1	2	3
Empirical formula	C_21_H_19_Cl_2_CuN_3_	C_21_H_19_Cl_3_CrN_3_	C_22_H_20.5_Cl_2_MnN_3.5_
Formula weight	447.83	471.74	459.76
Crystal system	Orthorhombic	Tetragonal	Triclinic
Space group	*Pbca*	*I*4_1_/*a*	*P* $\bar{1}$
*a* (Å)	15.632(8)	15.775(4)	8.988(3)
*b* (Å)	20.298(11)	15.775(4)	12.730(5)
*c* (Å)	25.070(13)	33.247(8)	20.143(7)
α (°)	90.00	90.00	80.318(8)
*β* (°)	90.00	90.00	79.252(7)
*γ* (°)	90.00	90.00	86.277(8)
*V* (Å^3^)	7955(7)	8274(3)	2230.7(14)
*Z*	16	16	4
*ρ*_calcd_ (Mg·m^−3^)	1.496	1.515	1.369
*μ* (mm^−1^)	1.377	0.953	0.845
*F*(000)	3664	3856	944
Crystal size (mm)	0.35 × 0.28 × 0.21	0.33 × 0.26 × 0.14	0.31 × 0.24 × 0.13
*θ* range for data collection (°)	2.31–18.37	1.43–25.10	2.07–25.10
Reflections collected	38,290	20,559	11,205
Independent reflections	7082	3688	7858
*R*_int_	0.1513	0.1302	0.0723
*Final R indices* [(*I* > 2*σ*(*I*)]	*R*_1_ = 0.0571 w*R*_1_ = 0.1052	*R*_1_ = 0.0613 w*R*_1_ = 0.1305	*R*_1_ = 0.0807 w*R*_1_ = 0.1638
*R indices* (all data)	*R*_2_ = 0.1291 w*R*_2_ = 0.1316	*R*_2_ = 0.1296 w*R*_2_ = 0.1651	*R*_2_ = 0.1985 w*R*_2_ = 0.2245
GOF	0.958	1.011	0.951

**Table 2:** Selected bond lengths (Å) and angles (°) for complexes **1**–**3**.

**1**					
Cu1―N1	2.121(4)	Cu1―Cl2	2.3857(19)	N2―C13	1.335(6)
Cu1―N2	1.950(5)	N1―C6	1.429(7)	N3―C14	1.277(6)
Cu1―N3	2.078(4)	N1―C7	1.296(7)	N3―C16	1.434(6)
Cu1―Cl1	2.2340(18)	N2―C9	1.337(6)		
Cu2―N4	2.107(4)	Cu2―Cl3	2.388(2)	N5―C34	1.324(6)
Cu2―N5	1.936(4)	N4―C27	1.429(7)	N6―C35	1.277(6)
Cu2―N6	2.086(4)	N4―C28	1.285(6)	N6―C37	1.435(6)
Cu2―Cl4	2.2076(18)	N5―C30	1.344(7)		
N1―Cu1―N3	155.34(18)	N2―Cu1―N3	77.64(18)	N3―Cu1―Cl2	95.23(13)
N1―Cu1―Cl1	100.62(13)	N2―Cu1―Cl1	141.29(13)	Cl1―Cu1―Cl2	109.23(7)
N1―Cu1―Cl2	94.55(13)	N2―Cu1―Cl2	109.45(13)		
N2―Cu1―N1	77.76(18)	N3―Cu1―Cl1	97.41(13)		
N4―Cu2―N6	154.87(17)	N5―Cu2―N6	77.50(18)	N6―Cu2―Cl3	98.07(13)
N4―Cu2―Cl4	101.51(13)	N5―Cu2―Cl4	149.59(14)	Cl4―Cu2―Cl3	106.88(7)
N4―Cu2―Cl3	93.57(13)	N5―Cu2―Cl3	103.48(14)		
N5―Cu2―N4	78.17(19)	N6―Cu2―Cl4	96.31(13)		
**2**					
Cl1―Cr1	2.3002(19)	Cr1―N2	2.001(5)	N2―C8	1.333(7)
Cl2―Cr1	2.3106(19)	Cr1―N3	2.111(5)	N2―C12	1.336(7)
Cl3―Cr1	2.3239(19)	N1―C6	1.441(7)	N3―C13	1.295(7)
Cr1―N1	2.109(5)	N1―C7	1.281(7)	N3―C14	1.448(7)
N1―Cr1―N3	153.68(19)	N2―Cr1―N3	77.1(2)	N3―Cr1―Cl2	88.06(14)
N1―Cr1―Cl1	100.10(14)	N2―Cr1―Cl1	176.62(16)	N3―Cr1―Cl3	89.96(14)
N1―Cr1―Cl2	90.44(14)	N2―Cr1―Cl2	87.15(14)	Cl1―Cr1―Cl2	92.43(7)
N1―Cr1―Cl3	89.14(14)	N2―Cr1―Cl3	87.58(14)	Cl1―Cr1―Cl3	92.88(7)
N2―Cr1―N1	76.55(19)	N3―Cr1―Cl1	106.21(14)	Cl2―Cr1―Cl3	174.67(8)
**3**					
Mn1―N1	2.186(6)	Mn1―Cl2	2.331(3)	N2―C7	1.278(10)
Mn1―N2	2.297(7)	N1―C9	1.350(10)	N3―C14	1.290(9)
Mn1―N3	2.303(7)	N1―C13	1.347(10)	N3―C16	1.417(8)
Mn1―Cl1	2.337(3)	N2―C6	1.449(10)		
Mn2―N5	2.200(7)	Mn2―Cl3	2.341(3)	N4―C28	1.280(10)
Mn2―N4	2.258(7)	N5―C30	1.352(10)	N6―C35	1.293(10)
Mn2―N6	2.271(7)	N5―C34	1.324(10)	N6―C37	1.451(10)
Mn2―Cl4	2.328(3)	N4―C27	1.445(10)		
N1―Mn1―N3	71.0(3)	N2―Mn1―N3	142.3(2)	N3―Mn1―Cl2	100.37(18)
N1―Mn1―Cl1	116.29(10)	N2―Mn1―Cl1	99.24(19)	Cl1―Mn1―Cl2	118.28(10)
N1―Mn1―Cl2	125.43(19)	N2―Mn1―Cl2	99.6(2)		
N2―Mn1―N1	71.3(3)	N3―Mn1―Cl1	98.97(18)		
N5―Mn2―N6	71.0(3)	N4―Mn2―N6	141.6(6)	N6―Mn2―Cl3	102.94(18)
N5―Mn2―Cl4	116.26(18)	N4―Mn2―Cl4	97.37(4)	Cl4―Mn2―Cl3	116.91(11)
N5―Mn2―Cl3	126.84(19)	N4―Mn2―Cl3	101.9(3)		
N4―Mn2―N5	70.3(3)	N6―Mn2―Cl4	96.89(4)		

**Table 3:** Conversion and turnover frequency (TOF) of various catalysts in the cycloaddition of CO_2_ to propylene oxide (PO).

Entry	Catalyst	Co-Catalyst	Conversion ^a^ (%)	TOF ^b^ (h^−^^1^)
1	none	DMAP	0	0
2	**1**	DMAP	43.1	216
3	**2**	DMAP	74.6	373
4	**3**	DMAP	62.3	312
5	**2**	none	0	0
6	none	TBAB	28.6	143
7	**1**	TBAB	40.8	204
8	**2**	TBAB	69.5	348
9	**3**	TBAB	61.4	307

Reaction conditions: temperature: 120 °C, pressure: 2.5 MPa, reaction time: 2 h, [PO] = 5.808 g, (100 mmol), [PO]/[Cat.]/[Co − cat.] = 1000:1:1 ^a^ The conversion obtained by internal standard method in GC analysis. ^b^ Turnover frequency (TOF) = moles of PO consumed per mole of catalyst per hour.

**Table 4:** Cycloaddition of PO to CO_2_ in the presence of complex **2**/DMAP under various conditions.

Entry	[PO]/[2]/[DMAP]	P (MPa)	Time (h)	Temp (°C)	Conversion ^a^ (%)	TOF ^b^ (h^−1^)
1	1000:1:1	2.0	2	120	67.3	337
2	1000:1:1	2.5	2	120	74.6	373
3	1000:1:1	3.0	2	120	82.8	429
4	1000:1:1	3.5	2	120	84.2	421
5	2000:1:1	2.5	2	100	69.5	695
6	2000:1:1	2.5	3	100	83.1	554
7	2000:1:1	2.5	4	100	86.7	434
8	2000:1:1	2.5	2	120	62.2	622
9	2000:1:1	2.5	1	200	51.3	1026
10	2000:1:1	2.5	1	220	41.6	832
11	3000:1:1	2.5	2	120	25.4	381
12	4000:1:1	2.5	2	120	14.8	296
13	2000:1:2	2.5	2	120	72.3	723
14	2000:1:4	2.5	2	120	15.3	153

Reaction conditions: [PO] = 11.62 g (0.2 mol). ^a^ The conversion obtained by internal standard method in GC analysis. ^b^ Turnover frequency (TOF) = moles of PO consumed per mole of catalyst per hour.

**Table 5:** Metal atom charge distribution, highest occupied molecular orbital (HOMO), and lowest unoccupied molecular orbital (LUMO) of complexes **1**–**3**.

Complex	Metal Atom Charge Distribution	LUMO/eV	HOMO/eV	ΔE/eV
**1**	0.460	−0.03090	−0.24552	0.21463
**2**	0.454	−0.15833	−0.16290	0.00457
**3**	0.697	−0.10583	−0.22280	0.11697

## Supplementary Materials

The following are available online. Figure S1: TG curves of complexes **1**–**3**; Figure S2: Infrared spectra of ligand(**L**) and complexes **1**–**3**; Figure S3: UV-Vis spectra of ligand (**L**) and complexes **1**–**3**; Figure S4: HOMO and LUMO frontier molecular orbitals of Complex **1**; Figure S5: HOMO and LUMO frontier molecular orbitals of Complex **2**; Figure S6: HOMO and LUMO frontier molecular orbitals of Complex **3**; Figure S7: Infrared spectra of catalytic product; Table S1: NBO charges for some atoms; Table S2:Selected bond lengths (Å) and angles (°) after optimizing for complexes **1**–**3**; X-ray molecular structure reported for complexes **1**–**3**. (CIF)
