# Supplementary material for: New Coordination Complexes Based on the 2,6-bis[1-(Phenylimino)ethyl] Pyridine Ligand: Effective Catalysts for the Synthesis of Propylene Carbonates from Carbon Dioxide and Epoxides
Source: Molecules. 2018 Sep 10;23(9):2304. doi: 10.3390/molecules23092304 (PMC6225293; doi:10.3390/molecules23092304)

checkCIF/PLATON report

Structure factors have been supplied for datablock(s) xb9805

THIS REPORT IS FOR GUIDANCE ONLY. IF USED AS PART OF A REVIEW PROCEDURE FOR PUBLICATION, IT SHOULD NOT REPLACE THE EXPERTISE OF AN EXPERIENCED CRYSTALLOGRAPHIC REFEREE.

No syntax errors found. CIF dictionary Interpreting this report

Datablock: xb9805

Bond precision: C-C = 0.0088 A Wavelength=0.71073

Cell:	a=15.632(8)	b=20.298(11)	c=25.070(13)
	alpha=90	beta=90	gamma=90
Temperature:	296 K		

	Calculated	Reported
Volume	7955(7)	7955(7)
Space group	P b c a	P b c a
Hall group	-P 2ac 2ab	-P 2ac 2ab
Moiety formula	C21 H19 Cl2 Cu N3	C21 H19 Cl2 Cu N3
Sum formula	C21 H19 Cl2 Cu N3	C21 H19 Cl2 Cu N3
Mr	447.84	447.83
Dx,g cm-3	1.496	1.496
Z	16	16
Mu (mm-1)	1.377	1.377
F000	3664.0	3664.0
F000’	3674.61	
h,k,lmax	18,24,29	18,24,29
Nref	7082	7082
Tmin,Tmax	0.632,0.748	0.642,0.745
Tmin’	0.613	

Correction method= # Reported T Limits: Tmin=0.642 Tmax=0.745
AbsCorr = MULTI-SCAN

Data completeness= 1.000 Theta(max)= 25.099

R(reflections)= 0.0571(3767) wR2(reflections)= 0.1316(7082)

S = 0.958 Npar= 491

The following ALERTS were generated. Each ALERT has the format
test-name_ALERT_alert-type_alert-level.
Click on the hyperlinks for more details of the test.

Alert level C

```
RINTA01_ALERT_3_C  The value of Rint is greater than 0.12
            Rint given   0.151
PLAT331_ALERT_2_C Small Average Phenyl C-C Dist C1       -C6            1.37 Ang.
PLAT331_ALERT_2_C Small Average Phenyl C-C Dist C16      -C21           1.37 Ang.
PLAT331_ALERT_2_C Small Average Phenyl C-C Dist C37      -C42           1.36 Ang.
PLAT341_ALERT_3_C Low Bond Precision on  C-C Bonds ...............   0.00882 Ang.
PLAT906_ALERT_3_C Large K Value in the Analysis of Variance ......     6.359 Check
PLAT978_ALERT_2_C Number C-C Bonds with Positive Residual Density.         0 Info
```

Alert level G

```
PLAT020_ALERT_3_G The Value of Rint is Greater Than 0.12 .........     0.151 Report
PLAT232_ALERT_2_G Hirshfeld Test Diff (M-X) Cu1      --Cl2       .       5.4 s.u.
PLAT794_ALERT_5_G Tentative Bond Valency for Cu1       (II)      .      2.25 Info
PLAT794_ALERT_5_G Tentative Bond Valency for Cu2       (II)      .      2.31 Info
PLAT910_ALERT_3_G Missing # of FCF Reflection(s) Below Theta(Min).         1 Note
PLAT933_ALERT_2_G Number of OMIT Records in Embedded .res File ...         1 Note
```

---

0 **ALERT level A** = Most likely a serious problem - resolve or explain
0 **ALERT level B** = A potentially serious problem, consider carefully
7 **ALERT level C** = Check. Ensure it is not caused by an omission or oversight
6 **ALERT level G** = General information/check it is not something unexpected

0 ALERT type 1 CIF construction/syntax error, inconsistent or missing data
6 ALERT type 2 Indicator that the structure model may be wrong or deficient
5 ALERT type 3 Indicator that the structure quality may be low
0 ALERT type 4 Improvement, methodology, query or suggestion
2 ALERT type 5 Informative message, check

It is advisable to attempt to resolve as many as possible of the alerts in all categories. Often the minor alerts point to easily fixed oversights, errors and omissions in your CIF or refinement strategy, so attention to these fine details can be worthwhile. In order to resolve some of the more serious problems it may be necessary to carry out additional measurements or structure refinements. However, the purpose of your study may justify the reported deviations and the more serious of these should normally be commented upon in the discussion or experimental section of a paper or in the "special_details" fields of the CIF. checkCIF was carefully designed to identify outliers and unusual parameters, but every test has its limitations and alerts that are not important in a particular case may appear. Conversely, the absence of alerts does not guarantee there are no aspects of the results needing attention. It is up to the individual to critically assess their own results and, if necessary, seek expert advice.

Publication of your CIF in IUCr journals

A basic structural check has been run on your CIF. These basic checks will be run on all CIFs submitted for publication in IUCr journals (*Acta Crystallographica*, *Journal of Applied Crystallography*, *Journal of Synchrotron Radiation*); however, if you intend to submit to *Acta Crystallographica Section C* or *E* or *IUCrData*, you should make sure that full publication checks are run on the final version of your CIF prior to submission.

Publication of your CIF in other journals

Please refer to the *Notes for Authors* of the relevant journal for any special instructions relating to CIF submission.

PLATON version of 14/07/2018; check.def file version of 05/06/2018

Datablock xb9805 - ellipsoid plot

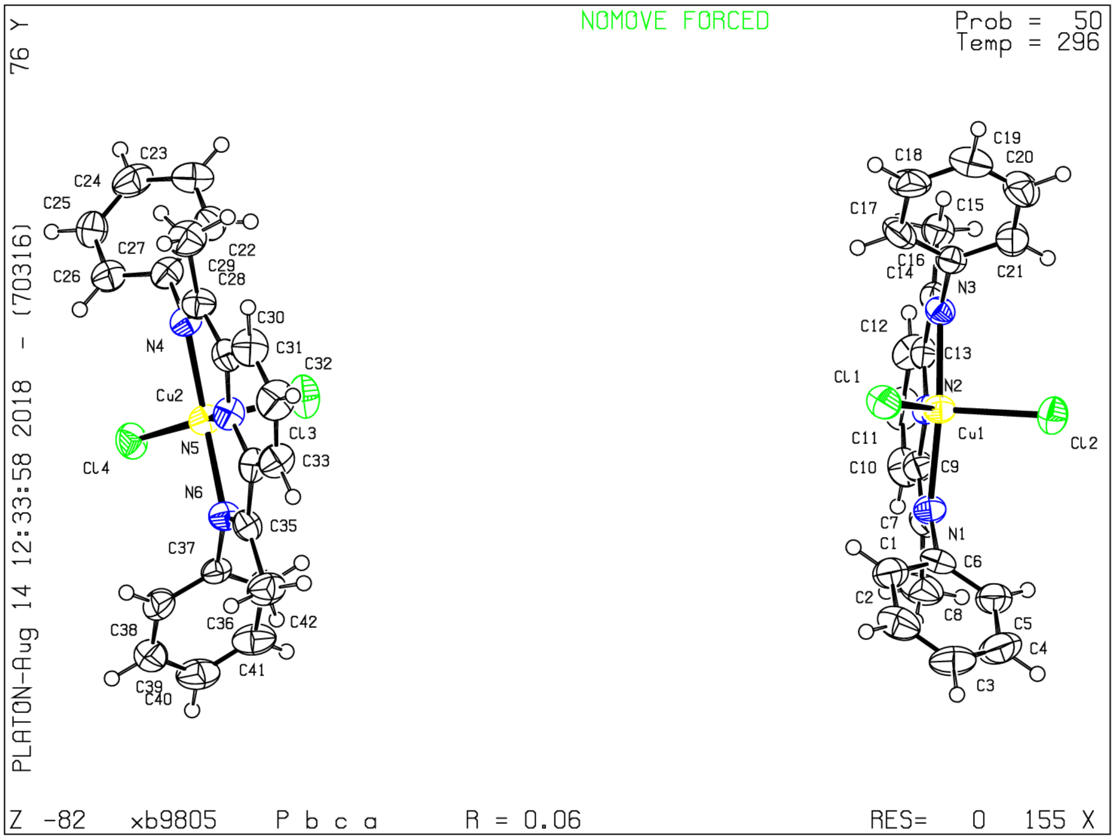

Supplement: Supplementary file 1 [file molecules-23-02304-s001.zip › Supplementary Materials/crystals data/complex1 Cu-checkcif.pdf]
