# Supplementary material for: New Coordination Complexes Based on the 2,6-bis[1-(Phenylimino)ethyl] Pyridine Ligand: Effective Catalysts for the Synthesis of Propylene Carbonates from Carbon Dioxide and Epoxides
Source: Molecules. 2018 Sep 10;23(9):2304. doi: 10.3390/molecules23092304 (PMC6225293; doi:10.3390/molecules23092304)

checkCIF/PLATON report

You have not supplied any structure factors. As a result the full set of tests cannot be run.

THIS REPORT IS FOR GUIDANCE ONLY. IF USED AS PART OF A REVIEW PROCEDURE FOR PUBLICATION, IT SHOULD NOT REPLACE THE EXPERTISE OF AN EXPERIENCED CRYSTALLOGRAPHIC REFEREE.

No syntax errors found.   CIF dictionary   Interpreting this report

Datablock: xb9697_0m

```
Bond precision:  C-C = 0.0102 A          Wavelength=0.71073

Cell:              a=15.775(4)      b=15.775(4)      c=33.247(8)
                   alpha=90         beta=90          gamma=90
Temperature:       296 K

                Calculated                 Reported
Volume          8274(5)                    8274(3)
Space group     I 41/a                     I4(1)/a
Hall group      -I 4ad                     ?
Moiety formula  C21 H19 Cl3 Cr N3          ?
Sum formula     C21 H19 Cl3 Cr N3          C21 H19 Cl3 Cr N3
Mr              471.74                     471.74
Dx,g cm-3       1.515                      1.515
Z               16                         16
Mu (mm-1)       0.953                      0.953
F000            3856.0                     3856.0
F000'           3869.08
h,k,lmax        18,18,39                   18,18,39
Nref            3688                       3688
Tmin,Tmax       0.743,0.875                0.743,0.877
Tmin'           0.730

Correction method= # Reported T Limits: Tmin=0.743 Tmax=0.877
AbsCorr = NONE

Data completeness= 1.000          Theta(max)= 25.100

R(reflections)= 0.0613( 2021)     wR2(reflections)= 0.1651( 3688)

S = 1.011                  Npar= 255
```

The following ALERTS were generated. Each ALERT has the format
test-name_ALERT_alert-type_alert-level.
Click on the hyperlinks for more details of the test.

Alert level C

```
ABSTY03_ALERT_1_C  The _exptl_absorpt_correction_type has been given as none.
            However values have been given for Tmin and Tmax. Remove
            these if an absorption correction has not been applied.
  From the CIF: _exptl_absorpt_correction_T_min   0.743
  From the CIF: _exptl_absorpt_correction_T_max   0.877
RINTA01_ALERT_3_C  The value of Rint is greater than 0.12
            Rint given   0.130
PLAT057_ALERT_3_C Correction for Absorption Required   RT(exp) ...       1.18 Do !
PLAT234_ALERT_4_C Large Hirshfeld Difference C1       --C2               0.16 Ang.
PLAT241_ALERT_2_C High   'MainMol' Ueq as Compared to Neighbors of         C3 Check
PLAT341_ALERT_3_C Low Bond Precision on  C-C Bonds ...............    0.01015 Ang.
PLAT601_ALERT_2_C Structure Contains Solvent Accessible VOIDS of .         42 Ang**3
```

Alert level G

```
PLAT005_ALERT_5_G No Embedded Refinement Details Found  in the CIF     Please Do !
PLAT020_ALERT_3_G The Value of Rint is Greater Than 0.12 .........      0.130 Report
PLAT066_ALERT_1_G Predicted and Reported Tmin&Tmax Range Identical          ? Check
PLAT083_ALERT_2_G SHELXL Second Parameter in WGHT  Unusually Large      15.19 Why ?
PLAT093_ALERT_1_G No s.u.'s on H-positions, Refinement Reported as      mixed Check
PLAT152_ALERT_1_G The Supplied and Calc. Volume s.u. Differ by ...          2 Units
PLAT432_ALERT_2_G Short Inter X...Y Contact  C11      ..C17              3.16 Ang.
                                           x,-1/2+y,-z  =     14_545 Check
PLAT710_ALERT_4_G Delete 1-2-3 or 2-3-4 Linear Torsion Angle ... #         13 Do !
             CL1 -CR1 -N2  -C8     10.00  3.00   1.555   1.555   1.555   1.555
PLAT710_ALERT_4_G Delete 1-2-3 or 2-3-4 Linear Torsion Angle ... #         18 Do !
             CL1 -CR1 -N2  -C12  -171.00  2.00   1.555   1.555   1.555   1.555
PLAT794_ALERT_5_G Tentative Bond Valency for Cr1       (III)     .       3.09 Info
PLAT899_ALERT_4_G SHELXL97   is Deprecated and Succeeded by SHELXL       2018 Note
```

```
   0 ALERT level A = Most likely a serious problem - resolve or explain
   0 ALERT level B = A potentially serious problem, consider carefully
   7 ALERT level C = Check. Ensure it is not caused by an omission or oversight
  11 ALERT level G = General information/check it is not something unexpected

   4 ALERT type 1 CIF construction/syntax error, inconsistent or missing data
   4 ALERT type 2 Indicator that the structure model may be wrong or deficient
   4 ALERT type 3 Indicator that the structure quality may be low
   4 ALERT type 4 Improvement, methodology, query or suggestion
   2 ALERT type 5 Informative message, check
```

It is advisable to attempt to resolve as many as possible of the alerts in all categories. Often the minor alerts point to easily fixed oversights, errors and omissions in your CIF or refinement strategy, so attention to these fine details can be worthwhile. In order to resolve some of the more serious problems it may be necessary to carry out additional measurements or structure refinements. However, the purpose of your study may justify the reported deviations and the more serious of these should normally be commented upon in the discussion or experimental section of a paper or in the "special_details" fields of the CIF. checkCIF was carefully designed to identify outliers and unusual parameters, but every test has its limitations and alerts that are not important in a particular case may appear. Conversely, the absence of alerts does not guarantee there are no aspects of the results needing attention. It is up to the individual to critically assess their own results and, if necessary, seek expert advice.

Publication of your CIF in IUCr journals

A basic structural check has been run on your CIF. These basic checks will be run on all CIFs submitted for publication in IUCr journals (*Acta Crystallographica*, *Journal of Applied Crystallography*, *Journal of Synchrotron Radiation*); however, if you intend to submit to *Acta Crystallographica Section C* or *E* or *IUCrData*, you should make sure that full publication checks are run on the final version of your CIF prior to submission.

Publication of your CIF in other journals

Please refer to the *Notes for Authors* of the relevant journal for any special instructions relating to CIF submission.

PLATON version of 14/07/2018; check.def file version of 05/06/2018

Datablock xb9697_0m - ellipsoid plot

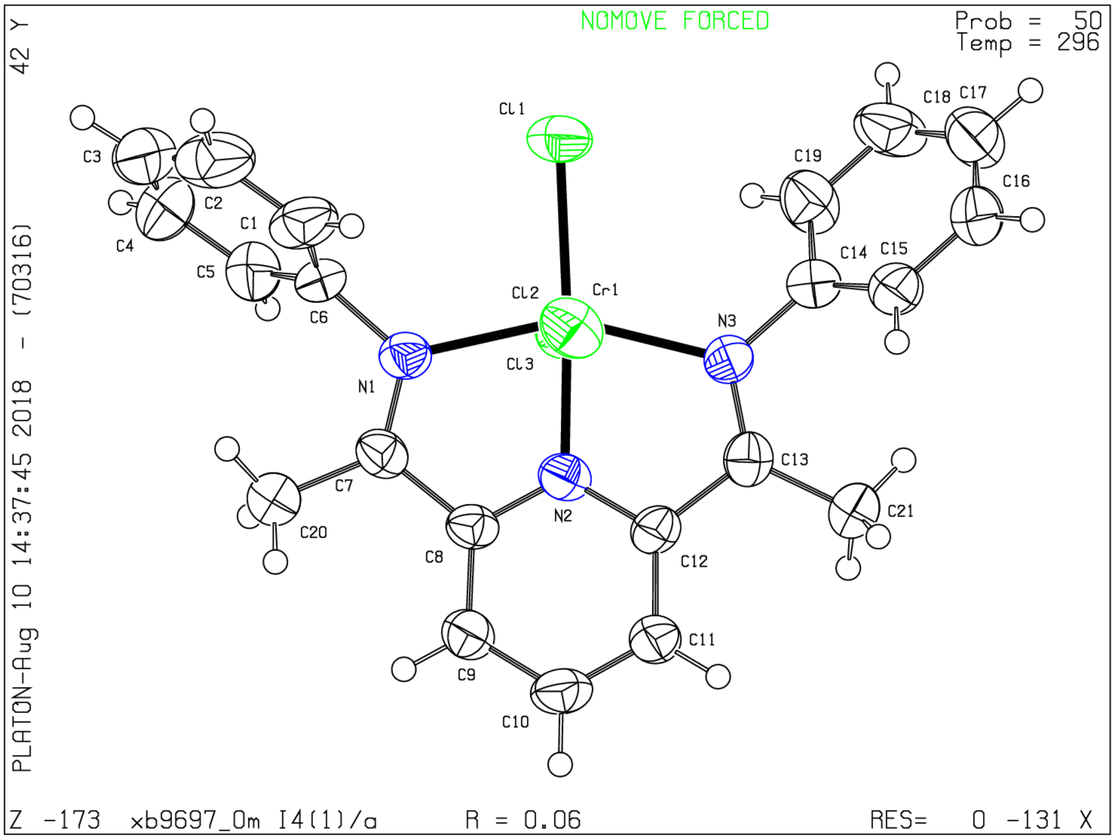

Supplement: Supplementary file 1 [file molecules-23-02304-s001.zip › Supplementary Materials/crystals data/complex2 Cr-checkcif.pdf]
