# Supplementary material for: New Coordination Complexes Based on the 2,6-bis[1-(Phenylimino)ethyl] Pyridine Ligand: Effective Catalysts for the Synthesis of Propylene Carbonates from Carbon Dioxide and Epoxides
Source: Molecules. 2018 Sep 10;23(9):2304. doi: 10.3390/molecules23092304 (PMC6225293; doi:10.3390/molecules23092304)

checkCIF/PLATON report

Structure factors have been supplied for datablock(s) xb9746_0m

THIS REPORT IS FOR GUIDANCE ONLY. IF USED AS PART OF A REVIEW PROCEDURE FOR PUBLICATION, IT SHOULD NOT REPLACE THE EXPERTISE OF AN EXPERIENCED CRYSTALLOGRAPHIC REFEREE.

No syntax errors found. CIF dictionary Interpreting this report

Datablock: xb9746_0m

Bond precision: C-C = 0.0136 A Wavelength=0.71073

Cell:	a=8.988(3)	b=12.730(5)	c=20.143(7)
	alpha=80.318(8)	beta=79.252(7)	gamma=86.277(8)
Temperature:	296 K		

	Calculated	Reported
Volume	2230.7(14)	2230.7(14)
Space group	P -1	P -1
Hall group	-P 1	-P 1
Moiety formula	2(C21 H19 Cl2 Mn N3), C2 H3 N	C21 H19 Cl2 Mn N3, 0.5(C2 H3 N)
Sum formula	C44 H41 Cl4 Mn2 N7	C22 H20.50 Cl2 Mn N3.50
Mr	919.52	459.76
Dx,g cm-3	1.369	1.369
Z	2	4
Mu (mm-1)	0.845	0.845
F000	944.0	944.0
F000’	946.74	
h,k,lmax	10,15,24	10,15,24
Nref	7938	7852
Tmin,Tmax	0.783,0.894	0.573,0.745
Tmin’	0.770	

Correction method= # Reported T Limits: Tmin=0.573 Tmax=0.745
AbsCorr = MULTI-SCAN

Data completeness= 0.989 Theta(max)= 25.100

R(reflections)= 0.0807(3202) wR2(reflections)= 0.2245(7852)

S = 0.951 Npar= 507

The following ALERTS were generated. Each ALERT has the format
test-name_ALERT_alert-type_alert-level.
Click on the hyperlinks for more details of the test.

Alert level C

```
CRYSC01_ALERT_1_C No recognised colour has been given for crystal colour.
PLAT018_ALERT_1_C _diffrn_measured_fraction_theta_max .NE. *_full          ! Check
PLAT026_ALERT_3_C Ratio Observed / Unique Reflections (too) Low ..       41% Check
PLAT234_ALERT_4_C Large Hirshfeld Difference C3       --C4              0.17 Ang.
PLAT234_ALERT_4_C Large Hirshfeld Difference C20      --C21             0.17 Ang.
PLAT234_ALERT_4_C Large Hirshfeld Difference C23      --C24             0.16 Ang.
PLAT244_ALERT_4_C Low    'Solvent' Ueq as Compared to Neighbors of       C44 Check
PLAT331_ALERT_2_C Small Average Phenyl C-C Dist C37      -C42           1.37 Ang.
PLAT341_ALERT_3_C Low Bond Precision on  C-C Bonds ...............   0.01359 Ang.
PLAT906_ALERT_3_C Large K Value in the Analysis of Variance ......    11.882 Check
PLAT906_ALERT_3_C Large K Value in the Analysis of Variance ......     2.587 Check
PLAT911_ALERT_3_C Missing FCF Refl Between Thmin & STh/L=    0.597        84 Report
PLAT978_ALERT_2_C Number C-C Bonds with Positive Residual Density.         0 Info
```

Alert level G

```
PLAT002_ALERT_2_G Number of Distance or Angle Restraints on AtSite         2 Note
PLAT003_ALERT_2_G Number of Uiso or Uij Restrained non-H Atoms ...         2 Report
PLAT042_ALERT_1_G Calc. and Reported MoietyFormula Strings  Differ    Please Check
PLAT045_ALERT_1_G Calculated and Reported Z Differ by a Factor ...      0.50 Check
PLAT172_ALERT_4_G The CIF-Embedded .res File Contains DFIX Records         1 Report
PLAT177_ALERT_4_G The CIF-Embedded .res File Contains DELU Records         1 Report
PLAT380_ALERT_4_G Incorrectly? Oriented X(sp2)-Methyl Moiety .....       C29 Check
PLAT794_ALERT_5_G Tentative Bond Valency for Mn1       (II)      .      2.16 Info
PLAT794_ALERT_5_G Tentative Bond Valency for Mn2       (II)      .      2.20 Info
PLAT860_ALERT_3_G Number of Least-Squares Restraints .............         2 Note
PLAT910_ALERT_3_G Missing # of FCF Reflection(s) Below Theta(Min).         3 Note
PLAT933_ALERT_2_G Number of OMIT Records in Embedded .res File ...         6 Note
```

---

0 **ALERT level A** = Most likely a serious problem - resolve or explain
0 **ALERT level B** = A potentially serious problem, consider carefully
13 **ALERT level C** = Check. Ensure it is not caused by an omission or oversight
12 **ALERT level G** = General information/check it is not something unexpected

4 ALERT type 1 CIF construction/syntax error, inconsistent or missing data
5 ALERT type 2 Indicator that the structure model may be wrong or deficient
7 ALERT type 3 Indicator that the structure quality may be low
7 ALERT type 4 Improvement, methodology, query or suggestion
2 ALERT type 5 Informative message, check

It is advisable to attempt to resolve as many as possible of the alerts in all categories. Often the minor alerts point to easily fixed oversights, errors and omissions in your CIF or refinement strategy, so attention to these fine details can be worthwhile. In order to resolve some of the more serious problems it may be necessary to carry out additional measurements or structure refinements. However, the purpose of your study may justify the reported deviations and the more serious of these should normally be commented upon in the discussion or experimental section of a paper or in the "special_details" fields of the CIF. checkCIF was carefully designed to identify outliers and unusual parameters, but every test has its limitations and alerts that are not important in a particular case may appear. Conversely, the absence of alerts does not guarantee there are no aspects of the results needing attention. It is up to the individual to critically assess their own results and, if necessary, seek expert advice.

Publication of your CIF in IUCr journals

A basic structural check has been run on your CIF. These basic checks will be run on all CIFs submitted for publication in IUCr journals (*Acta Crystallographica*, *Journal of Applied Crystallography*, *Journal of Synchrotron Radiation*); however, if you intend to submit to *Acta Crystallographica Section C* or *E* or *IUCrData*, you should make sure that full publication checks are run on the final version of your CIF prior to submission.

Publication of your CIF in other journals

Please refer to the *Notes for Authors* of the relevant journal for any special instructions relating to CIF submission.

PLATON version of 14/07/2018; check.def file version of 05/06/2018

Datablock xb9746_0m - ellipsoid plot

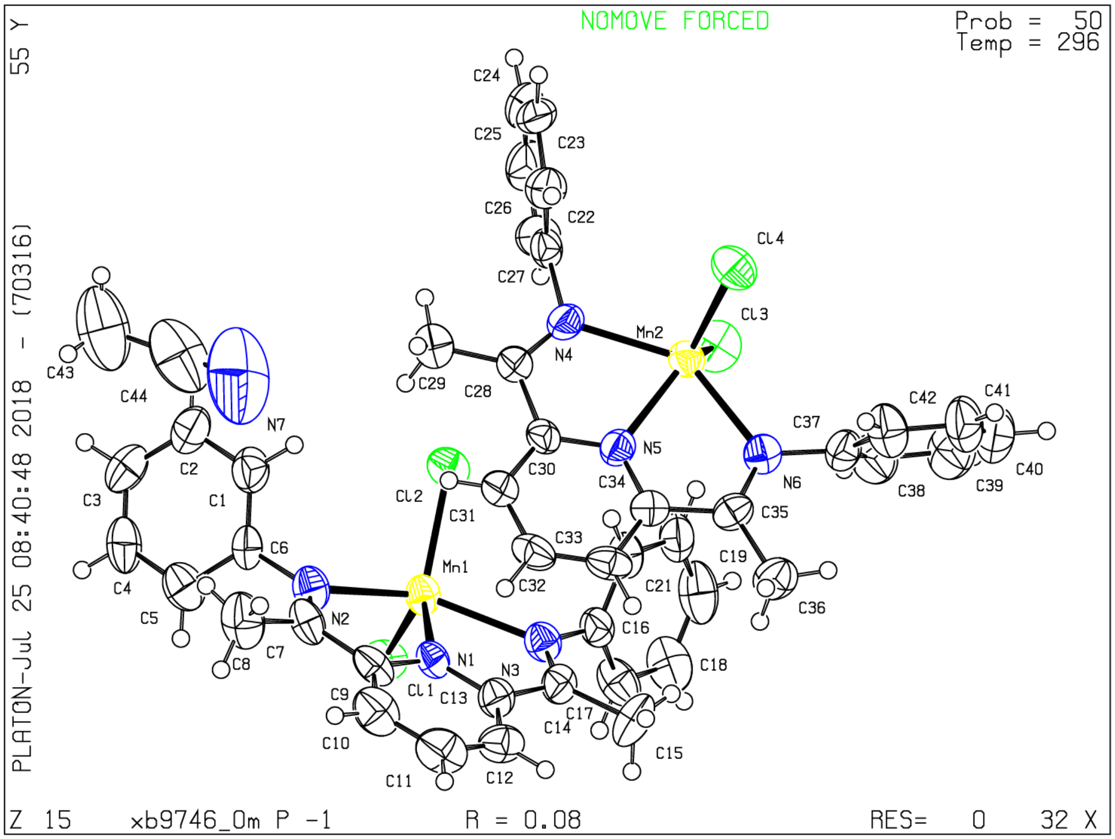

Supplement: Supplementary file 1 [file molecules-23-02304-s001.zip › Supplementary Materials/crystals data/complex3 Mn-checkcif.pdf]
