# Supplementary material for: New Coordination Complexes Based on the 2,6-bis[1-(Phenylimino)ethyl] Pyridine Ligand: Effective Catalysts for the Synthesis of Propylene Carbonates from Carbon Dioxide and Epoxides
Source: Molecules. 2018 Sep 10;23(9):2304. doi: 10.3390/molecules23092304 (PMC6225293; doi:10.3390/molecules23092304)
Supplement: Supplementary file 1 [file molecules-23-02304-s001.zip › Supplementary Materials/Supplementary Materials.doc]

**Supplementary Materials**

**New coordination compounds based on 2,6-bis[1-(phenylimino)ethyl] Pyridine** **ligand: effective catalysts for the synthesis of propylene carbonates from carbon dioxide and epoxides**

***Li Xia ^1^, Wen-Zhen Wang ^1*^_,_Shuang Liu ^1^, Xin-Gang Jia ^1^, Ying-Hui Zhang ^2^, Lei-Lei Li ^1^, Ya Wu ^1^, Bi-Yun Su ^1^, Shu-Bo Geng ^2^ and Wei Fan^1^***

***1.Thermogravimetric Analysis***

**2.Infrared spectrum characterization**

**3.Ultraviolet spectrum characterization**

***4.HOMO and LUMO frontier molecular orbitals of complexes (1*** – ***3)***

***5.The detailed quantum chemical calculation data***

***6.Selected bond lengths (Å) and angles (°) after optimizing***

***7.Infrared spectrum characterization of catalytic product***

***1.Thermogravimetric Analysis***

In order to detect the thermal stability of the complex, the thermal weight curve of complexes in the temperature range of 30 – 850℃ were determined in the N_2_ environment with a temperature rising rate of 15 /min, which is shown in Figure S1. The weight loss of complex **1** in the temperature range of 30-160℃ is small (3.68%), which corresponds to the lost of one free water molecule. It is consistent with the theoretical calculated value (3.85%). The complex **1** began to break down at 310℃ and the frame collapsed. The weight loss of complex **2** in the temperature range of 30 – 160℃ is small (7.44%), which corresponds to the lost of two free water molecules. It is consistent with the theoretical calculated value (7.69%). The complex **2** began to break down at 220℃. The weight loss of complex **3** in the temperature range of 30 – 130 ℃ is small (3.93%), which corresponds to the lost of one free water molecules. It is consistent with the theoretical calculated value (4.06%). The complex **3** began to break down at 240 ℃. From the thermogravimetric analysis, it is found that the decomposition temperature of **1** is the highest, subsequently followed by the complex **3** and complex **2**.

**Figure. S1** TG curves of complexes **1** – **3.**

**2.Infrared spectrum characterization**

The infrared spectra of the ligand L and the complex **1-3** are shown in Figure S2. The stretching vibration peak of the C-H bond near the aromatic ring of ligand L appears at 3000 cm^-1^. The aromatic ring stretching vibration peak (skeleton band) locates at the 1653~1492 cm^-1^. The out-of-plane bending vibration peak of the aromatic hydrogen appears at 955~633 cm^-1^. The aromatic ring stretching vibration peaks of complexes **1, 2** and **3** appear at 1637~1485 cm^-1^, 1637~1462 cm^-1^ and 1627~1485 cm^-1^, respectively. Compared with that of the ligand, these peaks move to the low frequencies by nearly 12 cm^-1^, 23 cm^-1^ and 17 cm^-1^, respectively, which indicate that the nitrogen atom of ligand coordinates with the metal ion. It is noteworthy here that infrared spectrum of Complex **2** shows the stretching vibration characterization of O-H around 3430 cm^-1^, probably due to the small amount of residual solvent methanol in Complex **2**.

**Fig. S2**  Infrared spectra of ligand(L) and complexes **1-3.**

**3.Ultraviolet spectrum characterization**

The UV spectra of ligand L and complexes **1-3** in CH_2_Cl_2_ solvent are shown in Figure S3, with the concentration being 0.005 g/L. The UV absorption peak of ligand L at 249 nm rea-shifted to 246 nm, 246 nm and 246 nm for complexes **1-3**, respectively, and the UV absorption peak of ligand L at 285 nm rea-shifted to 284 nm and 280 nm for complexes **1** and **3**, respectively. However, complex 2 does not appear absorption peak at 285nm. It may be caused by the electron transfer after the coordination between the central metal atom and ligand, and the π-π* transition of the complexes. The complex **3** has the most obvious redshift. And the molar absorption coefficient is the largest for complex **3**, while the smallest for complex **2**.

**Fig. S3** UV-Vis spectra of ligand (L) and complexes **1-3.**

***4.HOMO and LUMO frontier molecular orbitals of complexes (1 – 3)***

| (**a**) HOMO | (**b**) LUMO |
| --- | --- |

**Figure. S4** HOMO and LUMO frontier molecular orbitals of Complex **1**.

| (a) HOMO | (**b**) LUMO |
| --- | --- |

**Figure. S5** HOMO and LUMO frontier molecular orbitals of Complex **2**

| (a) HOMO | (b) LUMO |
| --- | --- |

**Figure. S6** HOMO and LUMO frontier molecular orbitals of Complex **3**

***5.NBO charges distribution of some atoms in complexes* 1** – **3.**

**Table S1** NBO charges for some atoms

| **1** | | **2** | | **3** | |
| --- | --- | --- | --- | --- | --- |
| atom | charge | atom | charge | atom | charge |
| Cu1 | 0.460 | Cr1 | 0.454 | Mn1 | 0.697 |
| N1 | -0.405 | N1 | -0.354 | N1 | -0.712 |
| N2 | -0.496 | N2 | -0.359 | N2 | -0.577 |
| N3 | -0.405 | N3 | -0.354 | N3 | -0.577 |
| C1 | -0.041 | C1 | -0.211 | C1 | -0.069 |
| C2 | -0.089 | C2 | -0.223 | C2 | -0.100 |
| C3 | -0.087 | C3 | -0.229 | C3 | -0.075 |
| C4 | -0.096 | C4 | -0.232 | C4 | -0.095 |
| C5 | -0.040 | C5 | -0.245 | C5 | -0.077 |
| C6 | 0.038 | C6 | 0.138 | C6 | 0.197 |
| C7 | 0.229 | C7 | 0.343 | C7 | 0.300 |
| C8 | -0.291 | C8 | 0.218 | C8 | -0.373 |
| C9 | 0.219 | C9 | -0.224 | C9 | 0.308 |
| C10 | -0.110 | C10 | 0.183 | C10 | -0.093 |
| C11 | 0.008 | C11 | -0.224 | C11 | 0.098 |
| C12 | -0.110 | C12 | 0.218 | C12 | -0.093 |
| C13 | 0.219 | C13 | 0.343 | C13 | 0.308 |
| C14 | 0.229 | C14 | 0.130 | C14 | 0.306 |
| C15 | -0.219 | C15 | 0.245 | C15 | -0.373 |
| C16 | 0.038 | C16 | 0.232 | C16 | 0.197 |
| C17 | -0.041 | C17 | -0.229 | C17 | -0.077 |
| C18 | -0.089 | C18 | -0.223 | C18 | -0.095 |
| C19 | -0.087 | C19 | -0.211 | C19 | -0.075 |
| C20 | -0.096 | C20 | -0.748 | C20 | -0.100 |
| C21 | -0.040 | C21 | -0.748 | C21 | -0.069 |
| Cl1 | -0.482 | Cl1 | -0.312 | Cl1 | -0.454 |
| Cl2 | -0.482 | Cl2 | -0.379 | Cl2 | -0.359 |
|  |  | Cl3 | -0.379 |  |  |

***6.Selected bond lengths (Å) and angles (°) after optimizing***

**Table S2**. Selected bond lengths (Å) and angles (°) after optimizing for complexes **1~3**

| **1** | | | | | |
| --- | --- | --- | --- | --- | --- |
| Cu1―N1 | 2.2203 | Cu1―Cl2 | 2.2999 | N2―C13 | 1.3254 |
| Cu1―N2 | 2.1566 | N1―C6 | 1.4197 | N3―C14 | 1.2815 |
| Cu1―N3 | 2.2201 | N1―C7 | 1.2815 | N3―C16 | 1.4197 |
| Cu1―Cl1 | 2.3000 | N2―C9 | 1.3254 |  |  |
| N1―Cu1―N3 | 147.5009 | N2―Cu1―N3 | 73.7529 | N3―Cu1―Cl2 | 91.8670 |
| N1―Cu1―Cl1 | 95.0567 | N2―Cu1―Cl1 | 102.5002 | Cl1―Cu1―Cl2 | 155.0121 |
| N1―Cu1―Cl2 | 91.8882 | N2―Cu1―Cl2 | 102.4876 |  |  |
| N2―Cu1―N1 | 73.7488 | N3―Cu1―Cl1 | 95.0757 |  |  |
| **2** | | | | | |
| Cl1―Cr1 | 2.3619 | Cr1―N2 | 2.0290 | N2―C8 | 1.3381 |
| Cl2―Cr1 | 2.2779 | Cr1―N3 | 2.1360 | N2―C12 | 1.3381 |
| Cl3―Cr1 | 2.3618 | N1―C6 | 1.4278 | N3―C13 | 1.2941 |
| Cr1―N1 | 2.1356 | N1―C7 | 1.2942 | N3―C14 | 1.4278 |
| N1―Cr1―N3 | 153.1790 | N2―Cr1―N3 | 76.5933 | N3―Cr1―Cl2 | 88.6593 |
| N1―Cr1―Cl1 | 103.4176 | N2―Cr1―Cl1 | 179.9960 | N3―Cr1―Cl3 | 89.1322 |
| N1―Cr1―Cl2 | 89.1284 | N2―Cr1―Cl2 | 85.2302 | Cl1―Cr1―Cl2 | 94.7724 |
| N1―Cr1―Cl3 | 88.6584 | N2―Cr1―Cl3 | 85.2266 | Cl1―Cr1―Cl3 | 94.7709 |
| N2―Cr1―N1 | 76.5857 | N3―Cr1―Cl1 | 103.4035 | Cl2―Cr1―Cl3 | 170.4668 |
| **3** | | | | | |
| Mn1―N1 | 1.9131 | Mn1―Cl2 | 2.2483 | N2―C7 | 1.3198 |
| Mn1―N2 | 2.0540 | N1―C9 | 1.3745 | N3―C14 | 1.3198 |
| Mn1―N3 | 2.0540 | N1―C13 | 1.3745 | N3―C16 | 1.4299 |
| Mn1―Cl1 | 2.3868 | N2―C6 | 1.4299 |  |  |
| N1―Mn1―N3 | 78.1624 | N2―Mn1―N3 | 154.5133 | N3―Mn1―Cl2 | 97.1309 |
| N1―Mn1―Cl1 | 102.6656 | N2―Mn1―Cl1 | 97.6774 | Cl1―Mn1―Cl2 | 108.4845 |
| N1―Mn1―Cl2 | 148.8499 | N2―Mn1―Cl2 | 97.1285 |  |  |
| N2―Mn1―N1 | 78.1634 | N3―Mn1―Cl1 | 97.6774 |  |  |

***7.Infrared spectrum characterization of catalytic product***

**Figure. S7**  Infrared spectra of catalytic product.
